# Fludarabine Treatment of Patient with Chronic Lymphocytic Leukemia Induces a Digital Ischemia

**DOI:** 10.1155/2016/7362791

**Published:** 2016-11-03

**Authors:** Utku Erdem Soyaltin, Deniz Yuce Yildirim, Mustafa Yildirim, Mehmet Can Ugur, Ferhat Ekinci, Cengiz Ceylan, Harun Akar

**Affiliations:** ^1^Tepecik Education and Research Hospital, Internal Medicine Clinic, Izmir, Turkey; ^2^Dortyol Public Hospital, Hatay, Turkey; ^3^Tepecik Education and Research Hospital, Hematology, Izmir, Turkey

## Abstract

We report a 63-year-old man with a history of chronic lymphocytic leukemia (CLL) who presented with asymmetrical Raynaud's phenomenon of sudden onset which progressed to acral gangrene rapidly in a week. These symptoms began approximately one week after the fourth cycle of fludarabine and cyclophosphamide chemotherapy and were accompanied by pain, numbness, and cyanosis in the fingers of his right hand except the first finger. Fludarabine may play a role in acral vascular syndrome. The treatment with fludarabine in patients with evolving digital ischemia should be carried out with caution.

## 1. Introduction

Acral ischemia or gangrene of sudden onset is rather uncommon and usually is seen in the setting of disseminated intravascular coagulation, sepsis, purpura fulminans, low cardiac output, collagen vascular disease, or antiphospholipid syndrome. It may rarely be seen in the malignant disorders either presenting sign, during the course of the disease, or in recurrence phase. In addition, digital ischemic events have been reported as adverse effects of several chemotherapeutic regimens including bleomycin and gemcitabine. In most patients, Raynaud's phenomenon becomes more severe with increasing dose and exposure. We report a 63-year-old man with a history of chronic lymphocytic leukemia (CLL) who presented with asymmetrical Raynaud's phenomenon of sudden onset which progressed rapidly to acral gangrene in a week under treatment of fludarabine and cyclophosphamide.

## 2. Case Report

**Figure 1:**
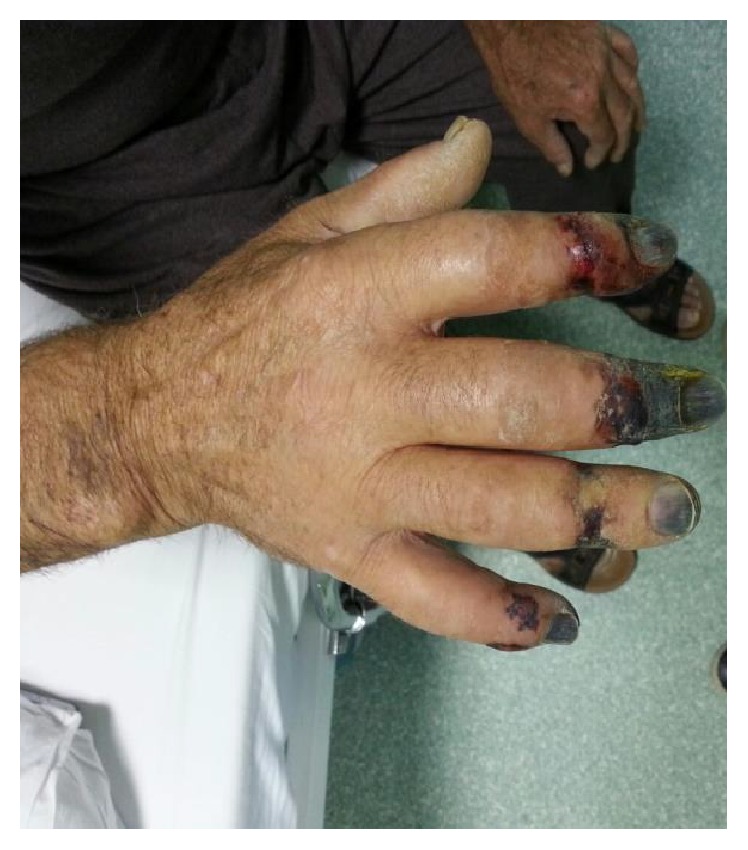


**Figure 2:**
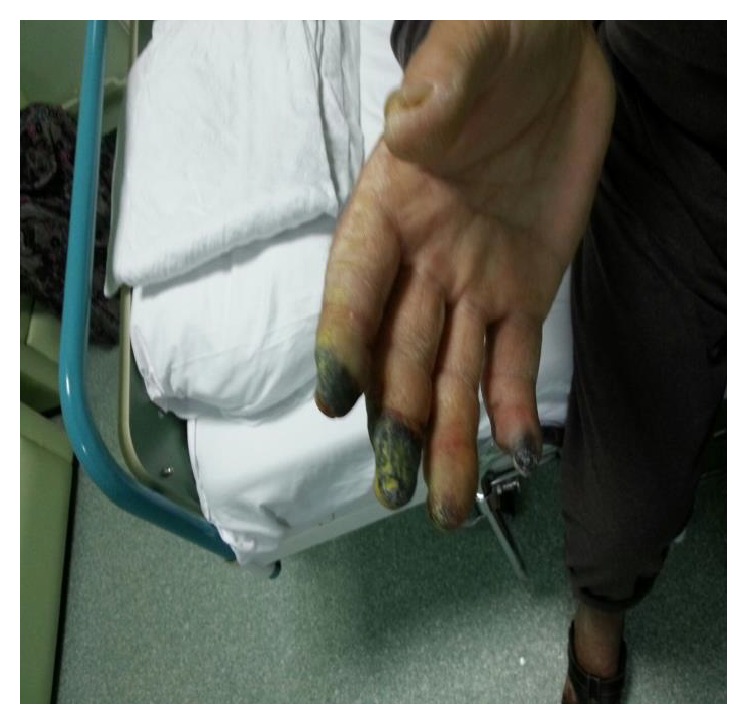


A 63-year-old man was diagnosed as CLL RAI stage 3 six moths ago and received four cycles of fludarabine and cyclophosphamide chemotherapy, because of 13q deletion (fludarabine 25 mg/m^2^ for 3 days every 28 days, cyclophosphamide 250 mg/m^2^ for 3 days). He has noticed a sudden onset of pain, numbness, and cyanosis in all right hand fingers except the first finger which started one week after the fourth cycle of the chemotherapy. Patient developed gangrenous changes of fingertips within one week. The time interval between the diagnosis of CLL and the appearance of digital acral necrosis was 4 months and one week. He denied tobacco and vasoconstrictive drug usage. He had no history of Raynaud's disease. Physical examination was remarkable for cyanosis and gangrene of all right hand fingertips except the first finger. The patient's vital signs were normal. Cardiac and pulmonary examination were within normal limits. Brachial and radial artery pulse was normal (Figures 1 and 2). Laboratory tests are shown in Table 1. ECG and chest X ray were normal. Arterial Doppler ultrasound of right upper extremities revealed patent arteries with normal flow pattern.

Nifedipine, aspirin, enoxaparin, and infusion of prostacyclin (PGI_2_) analogue were initiated. Since acral gangrene progressed under PGI_2_ infusion, hyperbaric oxygen therapy was initiated. Acral gangrene eventually stabilized in two weeks and second and third finger amputation was performed.

## 3. Discussion

Acrosyndromes associated with solid and hematological malignancies have been reported rarely. Raynaud's phenomenon occurring before digital gangrene in paraneoplastic disorders is rare [1]. However, history of heavy smoking, peripheral arterial occlusive disease (PAOD), dyslipidemia, hypertension, and diabetes mellitus during a diagnosis of cancer may already exist and cause ischemic events.

Poszepczynska-Guigné et al. described [2] two cases of paraneoplastic gangrene. In their literature review of the 68 patients determined as acral vascular syndromes, 40 reported having gangrene, 16 had acrocyanosis, and 12 had Raynaud's phenomenon. In their search, fingers were affected by 94% and adenocarcinomas were the predominantly associated malignancies (41%), and metastases were observed in 41%. In half of the patients, the acral vascular syndromes were found to be regressed after tumor treatment. The malignancies among patients with RP include the lung carcinoma, gastrointestinal carcinoma such as colon, pancreas, stomach, small intestine, kidney carcinoma, gynecological carcinoma such as uterus and ovary, and hematological malignancies such as myeloma, leukemia, and lymphoma [3, 4]. Among lymphoma-associated acral vascular syndromes, high grade lymphomas most frequently occur [5]. Also, there is a case report about a CLL patient which developed RP [6]. Primary RP predominantly affects young women, but paraneoplastic RP almost equally affects both sexes at an older age [7]. The progression of paraneoplastic Raynaud's phenomenon may be of short duration. In addition, it may be severe, with asymmetric involvement of the digits. More than 80% of the affected patients progress to ischemia, necrosis, pulp atrophy, and gangrene. Hypotheses of underlying pathogenetic mechanism between digital gangrene and malignancy are shown in the list in Section 3. Multiple mechanisms may be associated with the present case. It has been shown that arteritis of diverse etiologies is associated with digital artery vasospasm, as well as digital artery obstruction leading to finger ischemia and gangrene [8]. The underlying cause of the arteritis is unknown but is likely to be related to tumor antigen-antibody complexes with subsequent complement activation in the case of the contact with the arterial wall. The induction of vasculitis by antibodies to tumor antigens has been suggested as a possible mechanism [9]. It has been stated that digital ischemic symptoms improve after removal of renal and ovarian cancers in those with recurrence [9, 10]. Secondary RP has also been described in the context of combined chemotherapeutic regimens, especially those which contain bleomycin [11]. After three or four cycles of the treatment, 35% to 45% of patients developed persisting RP [12, 13]. A few case reports of gemcitabine-induced digital ischemia have been published [14]. Fludarabine can cause ischemic events but in literature we can find no digital ischemia secondary to fludarabine. The patient received four cycles of fludarabine and then Raynaud's phenomenon was noticed one week after chemotherapy. The mechanism of chemotherapy-induced Raynaud's phenomenon is not fully understood. It has been suggested that it may occur in the consequence of direct vascular toxicity with endothelial dysfunction due to chemotherapy or result from an abnormal sympathetic arterial vasoconstrictor response owing to neurotoxicity [12, 15]. Late treatment-associated morbidity due to peripheral neuropathy, including RP, has been observed in 20% to 40% of patients receiving chemotherapy [16].

 Hypotheses of pathogenetic mechanism of the association between digital gangrene and malignancy are as follows:

1. Sympathetic nerve invasion caused by cancer cells
2. Cryoglobulinemia secondary to carcinoma
3. Arteritis caused by cancer cells or their products
4. Vasculitis induced by antibodies against tumor antigens
5. Secretion of a vasoconstrictor substance by the neoplasm
6. Hyperviscosity
7. Hypercoagulability
8. Generalized vasospasm
9. Spontaneous platelet aggregation.

## 4. Conclusion

RP due to chemotherapy is rare. When it develops, it may be severe, requiring aggressive treatment. An immunological mechanism has been suggested. The fact that our patient presenting with sudden onset has rapidly progressive peripheral ischemia without other predisposing conditions suggests that there may be paraneoplastic digital ischemia secondary to fludarabine. A chemotherapeutic origin of acral vascular syndrome should also be taken into consideration in elderly patients in the absence of known causative factors. We found that hyperbaric oxygen and iloprost treatment were therapeutically useful options in our patient.

## Figures and Tables

**Table 1:** Summary of the laboratory tests.

C-reactive protein (crp)	0,516 mg/dL
Biochemical profile	Normal
CPK	46 U/L
Serum complement levels	C3: 59,7 mg/dL C4: 19,7 mg/dL
Rheumatoid factor	41,6 IU/L
Antinuclear antibodies	Negative
ANCA	Negative
Cryoglobulins	Negative
LDH	213 U/L
Hepatitis B surface antigen	Negative
Hepatitis C antibody	Negative
Anti-HIV	Negative
Antiphospholipid antibody	Negative
